# CD39/CD73 Dysregulation of Adenosine Metabolism Increases Decidual Natural Killer Cell Cytotoxicity: Implications in Unexplained Recurrent Spontaneous Abortion

**DOI:** 10.3389/fimmu.2022.813218

**Published:** 2022-02-10

**Authors:** Jianan Zhu, Guangmin Song, Xiaobo Zhou, Ting-Li Han, Xinyang Yu, Hao Chen, Toby Mansell, Boris Novakovic, Philip N. Baker, Richard D. Cannon, Richard Saffery, Chang Chen, Hua Zhang

**Affiliations:** ^1^Department of Obstetrics and Gynecology, The First Affiliated Hospital of Chongqing Medical University, Chongqing, China; ^2^Canada–China–New Zealand Joint Laboratory of Maternal and Fetal Medicine, Chongqing Medical University, Chongqing, China; ^3^The Chongqing Key Laboratory of Translational Medicine in Major Metabolic Diseases, The First Affiliated Hospital of Chongqing Medical University, Chongqing, China; ^4^Department of Obstetrics and Gynaecology, The Second Affiliated Hospital of Chongqing Medical University, Chongqing, China; ^5^Molecular Immunity, Murdoch Children’s Research Institute and Department of Paediatrics, University of Melbourne, Melbourne, VIC, Australia; ^6^College of Medicine, Biological Sciences and Psychology, University of Leicester, Leicester, United Kingdom; ^7^Department of Oral Sciences, Sir John Walsh Research Institute, Faculty of Dentistry, University of Otago, Dunedin, New Zealand; ^8^Institute of Life Sciences, Chongqing Medical University, Chongqing, China

**Keywords:** URSA, dNK, CD39, CD73, adenosine, TGF-β

## Abstract

Unexplained recurrent spontaneous abortion (URSA) is believed to be associated with impaired immunosuppression at the maternal-fetal interface, but the detailed molecular mechanism remains unclear. The ATP-adenosine metabolic pathway regulated by CD39/CD73 has recently been recognized to be important in immunosuppression. This study aimed to investigate the regulation of decidual natural killer (dNK) cells and fetal extravillous trophoblast (EVT) cells by CD39 and CD73 in URSA, as well as the possible regulatory mechanism of CD39/CD73 *via* the TGF-β-mTOR-HIF-1α pathway using clinical samples and cell models. Fewer CD39^+^ and CD73^+^ cells were found in the URSA decidual and villous tissue, respectively. Inhibition of CD39 on dNK cells transformed the cells to an activated state with increased toxicity and decreased apoptosis, and changed their cytokine secretion, leading to impaired invasion and proliferation of the co-cultured HTR8/SVneo cells. Similarly, inhibition of CD73 on HTR8/SVneo cells decreased the adenosine concentration in the cell culture media, increased the proportion of CD107a^+^ dNK cells, and decreased the invasion and proliferation capabilities of the HTR8/SVneo cells. In addition, transforming growth factor-β (TGF-β) triggered phosphorylation of mammalian target of rapamycin (mTOR) and Smad2/Smad3, which subsequently activated hypoxia-inducible factor-1α (HIF-1α) to induce the CD73 expression on the HTR8/SVneo cells. In summary, reduced numbers of CD39^+^ and CD73^+^ cells at the maternal-fetal interface, which may be due to downregulated TGF-β-mTOR-HIF-1α pathway, results in reduced ATP-adenosine metabolism and increased dNK cytotoxicity, and potentially contributes to URSA occurrences.

## Introduction

Unexplained recurrent spontaneous abortion (URSA) is defined as two or more consecutive spontaneous abortions (1, 2). The mechanism remains unclear, but is thought to be associated with immune intolerance. During early pregnancy in humans, the fetal extravillous trophoblast (EVT) cells invade into decidua and remodel the uterine spiral arteries (3). Decidua consists of epithelial cells and immune cells. Successful pregnancy requires a unique immune tolerant environment where delicate and complex crosstalk between the fetal-derived EVT cells and the maternal-derived decidual cells takes place (4).

Natural killer (NK) cells, a population of innate lymphoid cells, can lyse cancer cells and virus-infected cells. They play an important role in controlling the adaptive immune response by producing pro-inflammatory and anti-inflammatory cytokines (5, 6). Decidual natural killer (dNK) cells are a special type of NK cells, which constitutes 70% of immune cells in the decidua (7). The other 30% include macrophages, dendritic cells, and T cells (8). dNK cells are different from peripheral blood NK (pNK) cells, in that they are identified as CD56^bright^CD16^-^, while the pNK cells are mainly (95%) CD56^dim^CD16^+^ (7). The origin of dNK cells remains uncertain, but it is possible that they are from a group of minor agranular CD56^bright^CD16^-^ NK cells in the blood, which migrate into the uterus and transform (8). The role of dNK cells in the adaptive immune response, particularly the production of proinflammatory and anti-inflammatory cytokines, has been characterized (5, 6). It has been reported that dNK cells exhibit lower cytotoxicity, but higher secretion potential, than pNK cells in physiological settings (9–11). The dNK cells express a variety of surface receptors, such as NKp44 (CD336), NKp30 (CD337) and NKG2D (CD314) (1, 2). When these receptors are activated, an array of cytokines and growth factors that regulate the immune tolerance and angiogenesis at the maternal-fetal interface are produced, including GM-CSF, TNF-α, IFN-γ, IL-10, IL-8, IL-2, interferon-inducible protein-10 (IP-10), hepatocyte growth factor (HGF), PLGF, CCL-3, CCL-4 and several vascular endothelial growth factor (VEGF) family members (2, 9).

Adenosine and its phosphates ADP and ATP mediate several functions such as inflammation *via* binding to purine receptors on cell surfaces (12). Extracellular ATP and ADP concentrations are regulated by CD39 (NTPDase1) and CD73. CD39 is expressed on the extracellular surface of endothelial cells, particularly in human vascular and placental trophoblastic tissues (13). It is also expressed on the surface of certain immune cells such as neutrophils, monocytes, natural killer cells, and some subsets of T and B lymphocytes, where it inactivates nucleotides (12). CD39 hydrolyses ATP and ADP to produce AMP, and the membrane-bound ecto-5’-nucleotidase CD73 further hydrolyses AMP to produce adenosine (14, 15). Extracellular adenosine regulates the immune function of T lymphocytes (16–18), B lymphocytes (19–21) and NK cells (21–24) *via* binding to four different G protein–coupled purinergic receptors A1, A2A, A2B and A3. Thus, CD39 and CD73 can change pro-inflammatory immune cells driven by ATP to anti-inflammatory ones induced by adenosine (25). It has been demonstrated that CD39 and CD73 can mediate the growth and metastasis of tumor cells (24, 26–31). The adenosine effect mediated by CD39 and CD73 is considered one of the most important immunosuppressive regulatory pathways in the tumor microenvironment. Upregulated CD39/CD73 has been reported in a large number of solid cancer studies, and displays correlation with poor prognosis (32–35). Studies have shown that tumor cells and Treg cells co-express CD73 and CD39 and produce extracellular adenosine (36, 37). However, the effects of CD39 and CD73 on dNK and EVT cells, which are the main immune cells at the maternal-fetal interface maintaining immune tolerance, and the key cells for the remodeling of spiral arteries, respectively, have been seldom studied.

TGF-β is abundantly expressed in the endometrium and promotes its decidualization (38). It regulates the immunosuppression and immunoactivation balance *via* acting on type I (TbRI) and type II (TbRII) receptors that phosphorylate the downstream signal transducers Smad2 and Smad3 (39, 40). It also regulates the homeostasis of NK cells and inhibits their cytokine production and cytolytic activity (41, 42). TGF-β facilitates the transition of CD16^+^ pNK cells to CD16^-^ cells and inhibits the development and differentiation of human NK cells (43). It also transforms pNK cells into noncytotoxic and proangiogenic NK cells, a cell type similar to dNK cells, with the presence of hypoxia and a demethylating agent (34). In tumors, TGF-β signaling has been found to induce the generation of CD39/CD73 myeloid cells (44). However, dNK cells exist in a different microenvironment from pNK cells and exhibit a different phenotype, and it remains unknown whether TGF-β regulates CD39 and CD73 expression on dNK and EVT cells, respectively, at the maternal-fetal interface.

This study aimed to investigate the role of CD39/CD73 in the crosstalk between dNK and EVT cells at the maternal-fetal interface, and its relationship with URSA. We first measured CD39 and CD73 levels at the maternal-fetal interface of URSA and normal tissues, and then investigated the impact of CD39 and CD73 on the adenosine production and functions of dNK and EVT (or HTR8/SVneo) cells. Furthermore, the impact of TGF-β on the CD73 expression in HTR8/SVneo cells was explored to find a possible cause for the CD39/CD73 imbalance.

## Materials and Methods

### Clinical Sample Collection

Fresh decidual and villous samples were obtained at the First Affiliated Hospital of Chongqing Medical University from women with voluntary terminations of pregnancy (n = 30) or unexplained recurrent spontaneous abortions (URSA, n = 12). Abortions with genetic abnormalities detected by chorionic villus sampling or anatomical examination were excluded. Before the operation, informed consent from each participant was obtained. The study conformed to the Ethical Review Methods for Biomedical Research involving Humans adopted by the National Health Commission of the People’s Republic of China. All the samples (**Table S1**) were put in ice-cold phosphate buffer saline (PBS) in sterile containers after collection, and immediately transferred to the laboratory.

### Cell Lines

The human trophoblast HTR-8/SVneo cell line was purchased from the American Type Culture Collection (ATCC, USA), and cultured in Gibco™ RPMI 1640 medium (Thermo Fisher Scientific, USA) with L-glutamine, 10% fetal bovine serum (FBS, PAN-Biotech, Germany) and 1% penicillin-streptomycin. The K562 cells were purchased from the National Infrastructure of Cell Line Resource of China and cultured in the same medium. All the cells were grown in standard culture conditions (37°C and 5% CO_2_ in humidified air).

### Cell Preparation and Purification

The decidual tissue was first washed thoroughly with cold PBS, cut into pieces of about 1–3 mm, and digested by gentle shaking with 0.1% collagenase type IV (Catalog#: C5138, Millipore Sigma, USA) and 0.01% DNase I (Catalog#: 10104159001, Millipore Sigma, USA) for 1 h at 37°C. Afterwards, the mixture was sequentially filtered through nylon meshes of decreasing pore sizes (100, 200 and 400 mesh). The decidual mononuclear cells in the final filtrate were then concentrated by density gradient centrifugation (1000g, 20 min) with ficoll (Catalog#: 17144002, GE Healthcare, Sweden). After centrifugation, the mononuclear cells were collected, washed, and counted. The dNK cells from the decidual mononuclear cells were enriched using the human CD56^+^CD16^-^ NK Cell Isolation Kit (Catalog#: 130-092-661, Miltenyi Biotec, Germany) according to the manufacturer’s instructions. The isolated dNK cells were cultured in RPMI 1640 medium containing recombinant human IL-15 (10 ng/mL, Catalog#:I8648, Sigma), and then immediately underwent various analyses such as flow cytometry.

### Flow Cytometry Assays

The dNK cells were cultured in 6-well plates (5 × 10^5^ cells/well) for 24 hours. The mononuclear cells were resuspended in staining buffer, and then immediately stained with a range of monoclonal antibodies, namely anti-CD56 TULY56 FITC (Catalog#: 11-0566-42, eBioscience, USA), anti-CD16 PE (Catalog#: 12-0168-42, eBioscience, USA), anti-CD39 APC (Catalog#: 17-0399-41, eBioscience, USA), anti-CD107 APC H4A3 (Catalog#: 560664, BD Pharmingen, USA), anti-CD314 PE (Catalog#: 557940, BD Pharmingen, USA), anti-CD336 PE (Catalog#: 558563, BD Pharmingen, USA), anti-CD337 PE (Catalog#: 558407, BD Pharmingen, USA), anti-CD73 PE (Catalog#: 550257, BD Pharmingen, USA), anti-Annexin V FITC (Catalog#: 556419, BD Pharmingen, USA), anti-DAPI (Catalog#: 564907, BD Pharmingen, USA) and anti-CD45 APCCY7 (Catalog#: 348805, BD Pharmingen, USA). After incubation at room temperature for 30 min, the cells were then washed and resuspended in PBS for flow cytometry analysis (CytoFLEX, eBioscience, USA). The strategy for multidimensional flow cytometry analysis is shown in the **Supplementary Material, Figure S1**.

### ELISA

The concentrations of ATP (Catalog#: 14432H1, MEIMIAN) and adenosine (Catalog#: 1913H1, MEIMIAN) in the culture medium were determined with enzyme-linked immunosorbent assay (ELISA) Kits. The dNK cells were cultured in 24-well plates (2×10^5^ cells/well) for 24 hours. The culture medium was collected and centrifuged, and the supernatant was stored at –80°C until assayed according to the manufacturer’s protocols.

### Luminex Assay

The isolated dNK cells were cultured in 24-well plates (2×10^5^ cells/well) for 24 hours, and the medium was collected. A Luminex X200 System (Luminex, USA) was used to measure the cytokine levels in the medium. The cytokines TNF-α, IL-10, IL-4, IL-6, IL-8, IP-10, IL-5, IL-13, IFN-γ, IL-17, IL-1, IL-27, HGF, GM-CSF and Galectin-1were determined using human luminex discovery assays (LXSAHM-15). The concentrations were calculated based on the mean fluorescent intensity (MFI). Standard curves were generated for reference cytokines and used to calculate cytokine concentrations in the medium.

### Western Blot

The proteins in the villous and decidual tissues as well as the cultured cells were extracted using the RIPA lysis buffer (Catalog#: P0013B, Beyotime Biotechnology, China) with PMSF (Catalog#: ST506, 1mM, Beyotime Biotechnology, China). After separation with 10% SDS-PAGE, the proteins were transferred to a PVDF membrane. The membranes were blocked with TBST (TBS+Tween) containing 5% skimmed milk powder for 1 hour, and then incubated with the corresponding primary rabbit polyclonal antibodies in Primary Antibody Dilution Buffer (Catalog#: P0256, Beyotime Biotechnology, China) at 4°C overnight. The antibodies were CD39 (1:1000, Catalog#: ab223842, Abcam, UK), CD73 (1:1000, Catalog#: ab175396, Abcam, UK), TGF-β (1:2000, Catalog#: ab27969, Abcam, UK), Phospho-mTOR (1:1000, Catalog#: 5536, Cell Signaling Technology, USA), Phospho-Smad2 (1:1000, Catalog#: 3108, Cell Signaling Technology, USA), Phospho-Smad3 (1:1000, Catalog#: 9520, Cell Signaling Technology, USA), HIF-1α (1:1000, Catalog#: 36169, Cell Signaling Technology, USA) and β-actin (1:5000, Catalog#: GB11001, Servicebio, China). Then, the membranes were washed and incubated with HRP-conjugated secondary rabbit antibodies (1:10,000; Proteintech) at room temperature for 2 hours. Band signals were visualized and analyzed with enhanced chemiluminescent reagent (Millipore Sigma) and a Vilber Fusion image system (Fusion FX5 Spectra, France).

### RT-PCR

Total RNA was extracted from HTR8/SVneo cells using Invitrogen™ TRIzol reagent (Thermo Fisher Scientific, USA) according to the manufacturer’s instructions. cDNA was synthesized using Roche Reverse Transcription Kit (Catalog#: 07912455001, Roche), and qPCR was performed using the SYBR Premix Ex Taq (TaKaRa Biomedical Technology, China) with a LightCycler™ 96 instrument (Roche, Switzerland). GAPDH was used as the internal gene control. Its primer pair was: forward: 5′CAGGAGGCATTGCTGATGAT3′, reverse: 5′GAAGGCTGGGGCTCATTTT3′. The primer pairs of the tested genes were as follows: CD73 forward: 5′ACCAGCGAGGACTCCAGCAAG3′, reverse: 5′AGCAGCAGCACGTTGGGTTC3′, HIF-1α forward: 5′ATCAGACACCTAGTCCTTCCGATGG3′, reverse: 5′GTGGTAGTGGTGGCATTAGCAGTAG3′.

### Immunohistochemistry

The villous and decidual tissues were first cut into 4 μm-thick slices. Immunohistochemical staining was then performed according to standard procedures (45). Primary antibodies for TGF-β (1:200, Catalog#: ab27969, Abcam), cytokeratin 7 (1:200, Catalog#: ET1609-62, HUABIO), CD39(1:200, Catalog#: ab223842, Abcam), CD73 (1:200, Catalog#: ab175396, Abcam), or NCAM (1:200, Catalog#: ET1702-43, HUABIO) was added and incubated overnight. The antibodies were quantified with 3,3’-diaminobenzidine (DAB) staining (ZSGB Biotech, China). ImageJ software was used to measure the average optical density (AOD) of positive signal in each view field.

### Cell Invasion Assay

Transwell™ inserts (8 µm) containing polycarbonate membranes (Catalog#: 3428, Corning Incorporated, USA) were coated with an 8× dilution of Matrigel (Catalog#: 356234, Corning Incorporated, USA). HTR8/SVneo cells (5×10^4^) were seeded into the upper chamber with serum‐free culture medium and the isolated dNK cells (1×10^5^) were placed in the lower chamber with culture medium supplemented with 10% FBS, and incubated for 24 hours. The cells were stained with crystal violet and observed with light microscopy (EVOS FL Auto Imaging System, Thermo Fisher Scientific, USA). The invasion rate was measured with the ImageJ software.

### Cell Proliferation Assay and Measurement of Mitochondrial Membrane Potential

Approximately 5×10^3^ HTR8/SVneo cells and 1×10^4^ isolated dNK cells were co-cultured in the central part of each well in a 96-well plate for 24 hours. Then, the suspended dNK cells were discarded and fresh culture medium was added. After 24-hour incubation, 10 μL of CCK-8 solution was added into each well, and the plates were incubated for another 2 hours. A microplate reader (Thermo Fisher Scientific, USA) was used to measure the absorbance at 450 nm of each well.

Mitochondrial membrane potential (ΔΨm) is an important parameter of mitochondrial function (46, 47). Loss of ΔΨm is a sign of early apoptosis, and hence was also measured in dNK cells using the cationic probe JC-1 (Catalog#: C2006, Beyotime Biotechnology, China) according to the manufacturer’s instructions. In short, the isolated dNK cells were cultured in 12-well plates (2×10^5^ cells/well) for 12 hours. Then the cells were collected and incubated with JC-1 staining solution (5 μg/ml) at 37°C for 20 min. The cells were then washed twice with JC-1 staining buffer, and the fluorescence intensities of the mitochondrial JC-1 monomers (λex=514 nm, λem=529 nm) and aggregates (λex=585 nm, λem=590 nm) were measured with a fluorescence microscope (EVOS FL Auto Imaging System, Thermo Fisher Scientific, USA) and a flow cytometer (CytoFLEX, eBioscience, USA), respectively. The ΔΨm were calculated as the fluorescence ratio of red (i.e., aggregate) to green (i.e., monomer) signals.

### Statistical Analysis

Statistical analysis was carried out with GraphPad Prism (version 8, GraphPad Software, USA). Comparisons between two groups were performed using unpaired two-tailed t tests, and those among three or more groups were using one-way analysis of variance (ANOVA). All data are presented as mean ± SEM; P < 0.05 was considered statistically significant.

## Results

### Lower Levels of CD39 and CD73 Were Found in URSA Patients

**Figure 1:**
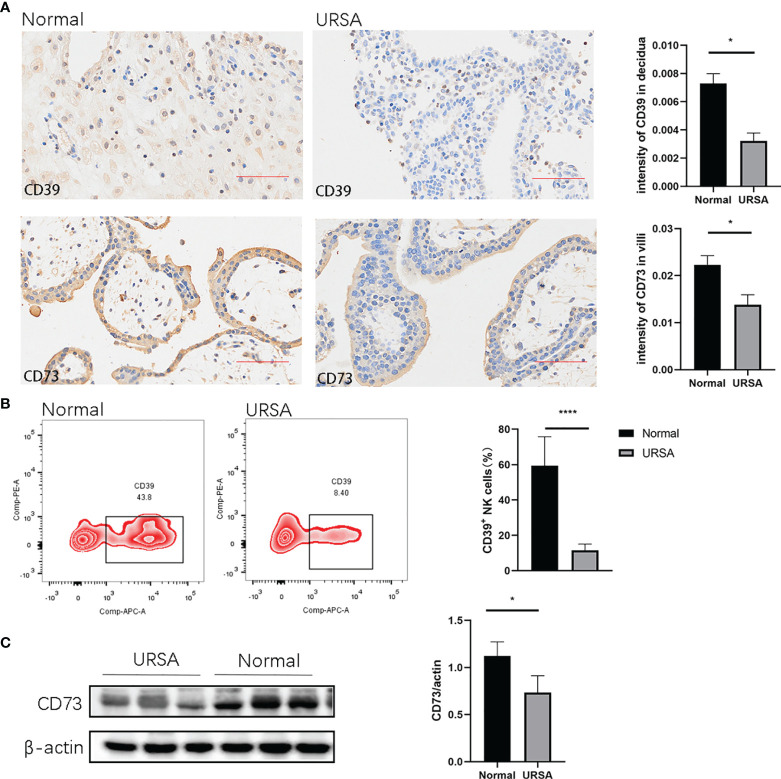
Expression of CD39 and CD73 in normal pregnancy and URSA patients. **(A)** Immunohistochemical staining of CD39 in decidual tissues and CD73 in villous tissues in normal pregnancies (n=3) and URSA patients (n=3). **(B)** Percentages of CD39^+^ dNK cells in the decidual tissues of normal pregnant women (n=6) and URSA patients (n=6). **(C)** Western blot analyses of CD73 in the villous tissue of normal pregnant women (n=3) and URSA patients (n=3). Results are shown as mean ± SEM, *P < 0.05, ****P < 0.0001 by two-tailed t tests. AOD, average optical density.

To assess the expression of CD39 and CD73 in normal pregnancy and URSA patients, we performed immunohistochemical staining analysis. It was found that CD39 was present in the dNK cells that also express CD56, and CD73 was present in trophoblast cells that also express CK7 (**Figures 1A** and **S2**). The intensity values of CD39 in the decidua and those of CD73 in the villi from the URSA patients were both significantly lower than those from normal pregnancies (**Figure 1A**). Since there are multiple types of immune cells in the decidual tissue expressing CD39, we further used multicolor flow cytometry to measure the proportions of CD39^+^ dNK cells in the decidua of normal pregnancies and URSA patients. The proportions of CD39^+^ dNK cells in URSA were significantly lower than those of normal specimens (**Figure 1B**). Western blot analysis also showed that the levels of CD73 in villous tissues of URSA patients were significantly lower than those of normal pregnancies (**Figure 1C**).

### Impacts of CD39/CD73 on the Extracellular Levels of ATP and Adenosine

**Figure 2:**
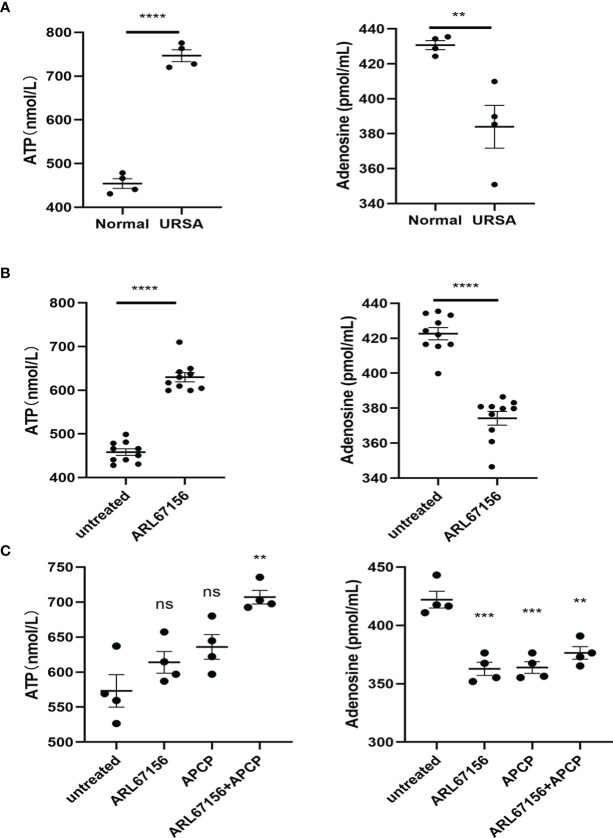
Production of ATP and adenosine in dNK cells. **(A)** dNK cells were isolated from decidual tissues of normal pregnant women (n=4) and URSA patients (n=4), and then cultured in a 24-well plate (2 × 10^5^ cells/well) for 24 hours. The concentrations of ATP and adenosine in the media were measured using ELISA (mean ± SEM, ****P < 0.0001, two-tailed t tests). **(B)** Freshly prepared dNK cells (n=10) were isolated from healthy decidua and incubated in the absence or presence of CD39 inhibitor (ARL67156) in a 24-well plate (2 × 10^5^ cells/well) for 24 hours. The concentrations of ATP and adenosine in the media were measured using ELISA (mean ± SEM, **P < 0.01, ****P < 0.0001, two-tailed t tests). **(C)** dNK cells isolated from healthy decidua (n=4) were co-cultured with HTR-8/SVneo cells (dNK cells:HTR-8/SVneo cells = 2:1) and incubated in the absence or presence of the CD39 inhibitor ARL67156 and/or the CD73 inhibitor APCP in a 24-well plate (2 × 10^5^ cells/well) for 24h. The concentrations of ATP and adenosine in the media were measured using ELISA (mean ± SEM, ns, statistically not significant, **P < 0.01, ***P < 0.001 compared with the untreated group, one-way ANOVA and *post hoc* tests).

We first isolated dNK cells from the decidual tissue of normal pregnant women and URSA patients, and cultured them *in vitro* for 24 hours. The concentrations of ATP and adenosine in the media were determined using ELISA. The concentration of ATP was found to be significantly higher, while that of adenosine was significantly lower in the URSA medium, indicating a hampered metabolism from ATP to adenosine, which was catalyzed by CD39 on dNK cells from the URSA patients (**Figure 2A**).

We then further analyzed whether the abovementioned effects were related to CD39 of the dNK cells by adding the CD39 inhibitor ARL67156 into the media of dNK cells from normal pregnancies. Isolated dNK cells were cultured in the absence or presence of the CD39 inhibitor for 24 hours. Results showed the CD39 inhibitor increased the concentration of ATP and decreased that of adenosine (**Figure 2B**).

Additionally, we investigated the CD39/CD73 regulation on ATP and adenosine production at the maternal-fetal interface by co-culturing dNK and HTR8/SVneo cells, with or without ARL67156 and the CD73 inhibitor APCP. In this co-culture system, both individual and combinational addition of ARL67156 and APCP significantly attenuated adenosine production and increased ATP concentration (**Figure 2C**).

### Impacts of CD39/CD73 on the Apoptosis and Mitochondrial Function of Activated dNK Cells

**Figure 3:**
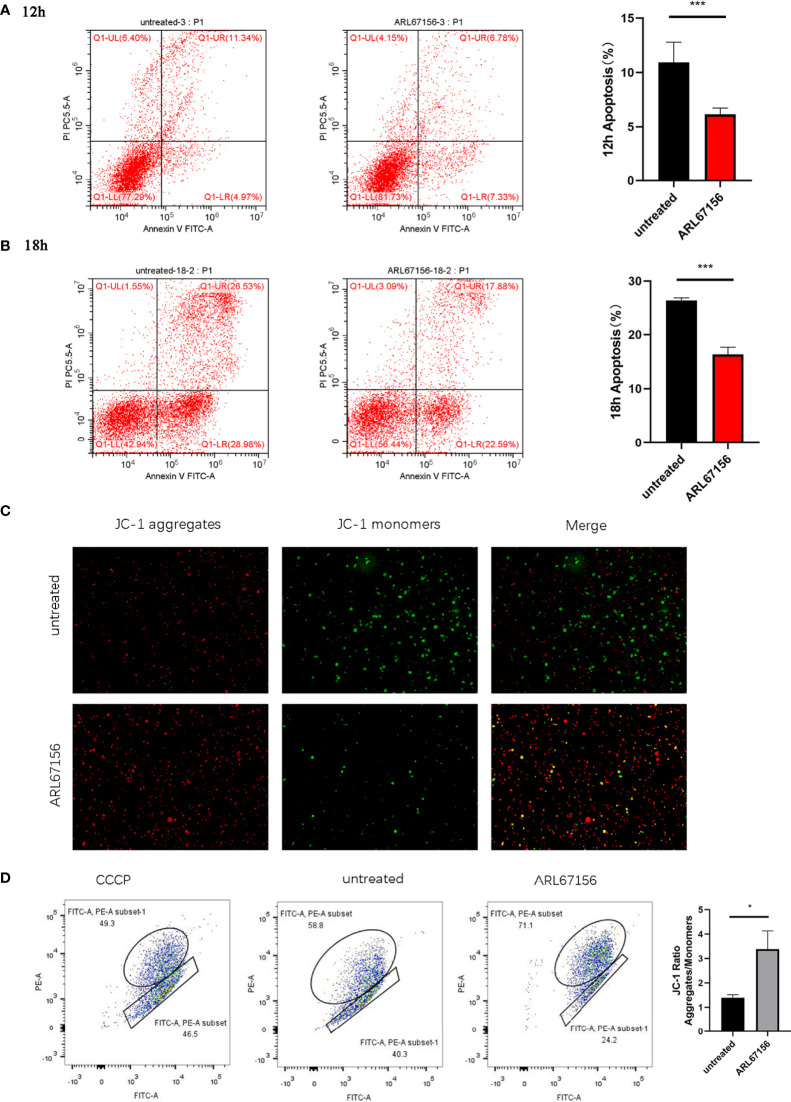
The effect of CD39/CD73 inhibiting on the apoptosis and mitochondrial membrane potential (ΔΨm) of dNK cells. dNK cells were isolated from healthy decidua of first-trimester pregnancies (n = 3), incubated in the absence or presence of the CD39 inhibitor ARL67156 for 12 hours **(A)** or 18 hours **(B)**, and then double stained with Annexin V and DAPI (mean ± SEM, ***P < 0.001, two-tailed t tests). **(C)** Microscope images taken from the JC-1 monomer fluorescence channel (green) and aggregate fluorescence channel (red) of dNK cells (n=5) with and without incubation with ARL67156 for 12 h. The monomeric JC-1 form was excited using a 525 nm laser, observed at an emission wavelengths of 514~529 nm, and is shown in green. The aggregate form was excited using a 566 nm laser, observed at 585~590 nm, and is shown in red. **(D)** Flow cytometry-based JC-1 assay as a measure of changes in mitochondrial membrane potential in dNK cells (n=5) induced by the CD39 inhibitor. The upper left quadrant indicates the cells with more JC-1 as aggregates with red fluorescence (i.e., normal ΔΨm), the lower right quadrant indicates the cells with more JC-1 as monomers with green fluorescence (i.e. low ΔΨm). (mean ± SEM, *P < 0.05 compared with the untreated group).

We investigated the effect of CD39 on dNK cell apoptosis by culturing the activated dNK cells with or without the CD39 inhibitor ARL67156 for 12 or 18 hours, followed by staining with Annexin V and DAPI. The percentages of Annexin V^+^ DAPI^+^ dNK cells cultured with CD39 inhibitors were found to be lower than those untreated (**Figure 3**). This indicates that CD39 promotes apoptosis of dNK cells. We further investigated the effect of CD39 on ΔΨm of the dNK cells, the loss of which is an indicator of dNK apoptosis. It was observed that the JC-1 red/green ratio was higher in dNK cells treated with CD39 inhibitors, suggesting reduced ΔΨm caused by CD39.

### Impacts of CD39/CD73 on Cytokine Secretion by dNK Cells

**Figure 4:**
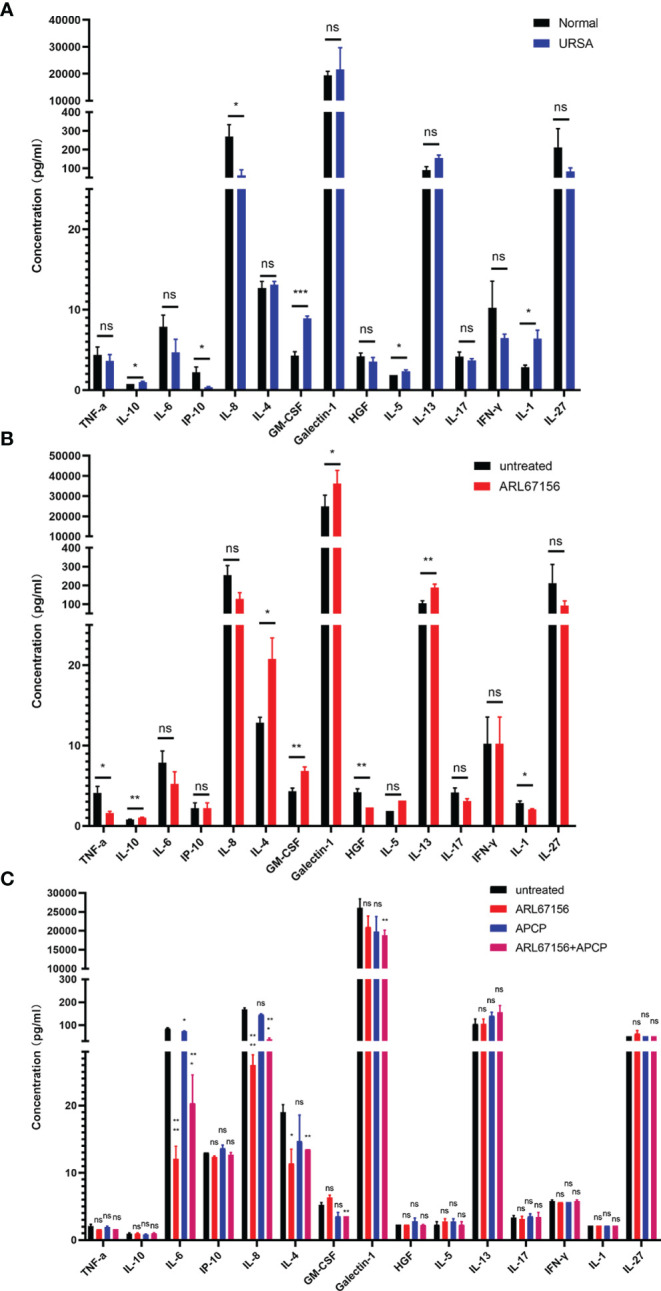
The effect of CD39/CD73 inhibiting on the cytokine secretion of dNK cells. **(A)** dNK cells were isolated from decidual tissues of normal pregnant women (n=4) and URSA patients (n=4), and cultured in a 24-well plate (2 × 10^5^ cells/well) for 24 hours. The cytokine concentrations in the media were determined using Luminex assay (mean ± SEM, ns, statistically not significant, *P < 0.05, ***P < 0.001, two-tailed t tests). **(B)** Cytokine concentrations in the media of dNK cells (n=5) cultured in the absence or presence of the CD39 inhibitor ARL67156 in 24-well plates for 24 hours (mean ± SEM, ns, statistically not significant, *P < 0.05, **P < 0.01, two-tailed t tests). **(C)** Cytokine concentrations in the media at 24 hours of co-culture of dNK and HTR-8/SVneo cells (n=3) (mean ± SEM, ns, statistically not significant, *P < 0.05, **P < 0.01, ***P < 0.001, ****P < 0.0001 compared with the untreated group, one-way ANOVA and *post hoc* tests).

We first evaluated whether the cytokine secretions of dNK cells from normal pregnant women and those from URSA patients were different. The dNK cells isolated from the decidual tissues of both donor groups were cultured for 24 hours, and then the cytokine concentrations in the culture media were determined using a multiplex cytokine assay. The dNK cells from URSA patients secreted significantly higher amounts of GM-CSF, IL-1, IL-5 and IL-10, with a trend of more IL-13, but lower levels of IL-8 and IP-10, and a trend of less HGF, IFN-γ, IL-6, IL-17 and IL-27 than dNK cells from the normal group. There was no difference in the secretion of IL-4 and TNF-α between the two groups (**Figure 4A**).

Next, we isolated dNK cells from normal pregnancies, and cultured them in the absence or presence of the CD39 inhibitors ARL67156. The cytokines in the culture media were then analyzed. The results showed that dNK cells cultured with the CD39 inhibitor secreted significantly more IL-10, IL-4, GM-CSF, galectin-1 and IL-13, with a trend of more IL-5 than the untreated group. The secretion of TNF-α, HGF and IL-1, however, was suppressed in cells treated with the CD39 inhibitor, and IL-6, IL-8, IL-17 and IL-27 displayed a trend of decreased secretion. Little or no effect on the secretion of IP-10, IL-8 and IFN-γ was observed (**Figure 4B**).

Furthermore, we explored the influences of CD39 and CD73 on cytokine secretion in the co-culture system. Interestingly, co-cultivation of dNK and HTR-8/SVneo cells in the presence of only the CD39 inhibitor or both the CD39 and CD73 inhibitors decreased the secretion of IL-6, IL-8 and IL-4, but the CD73 inhibitor alone did not significantly affect the secretion of these cytokines. The secretion of GM-CSF and galectin-1 was significantly reduced only in the presence of both the CD39 and CD73 inhibitors. There was no significant difference in the secretion of TNF-α, IL-10, IP-10, HGF, IL-5, IL-13, IL-17, IFN-γ, IL-1 or IL-27 when the co-culture system was treated with either the CD39 or CD73 inhibitor (**Figure 4C**).

### Impacts of CD39/CD73 on the Cytotoxicity of Activated dNK Cells

**Figure 5:**
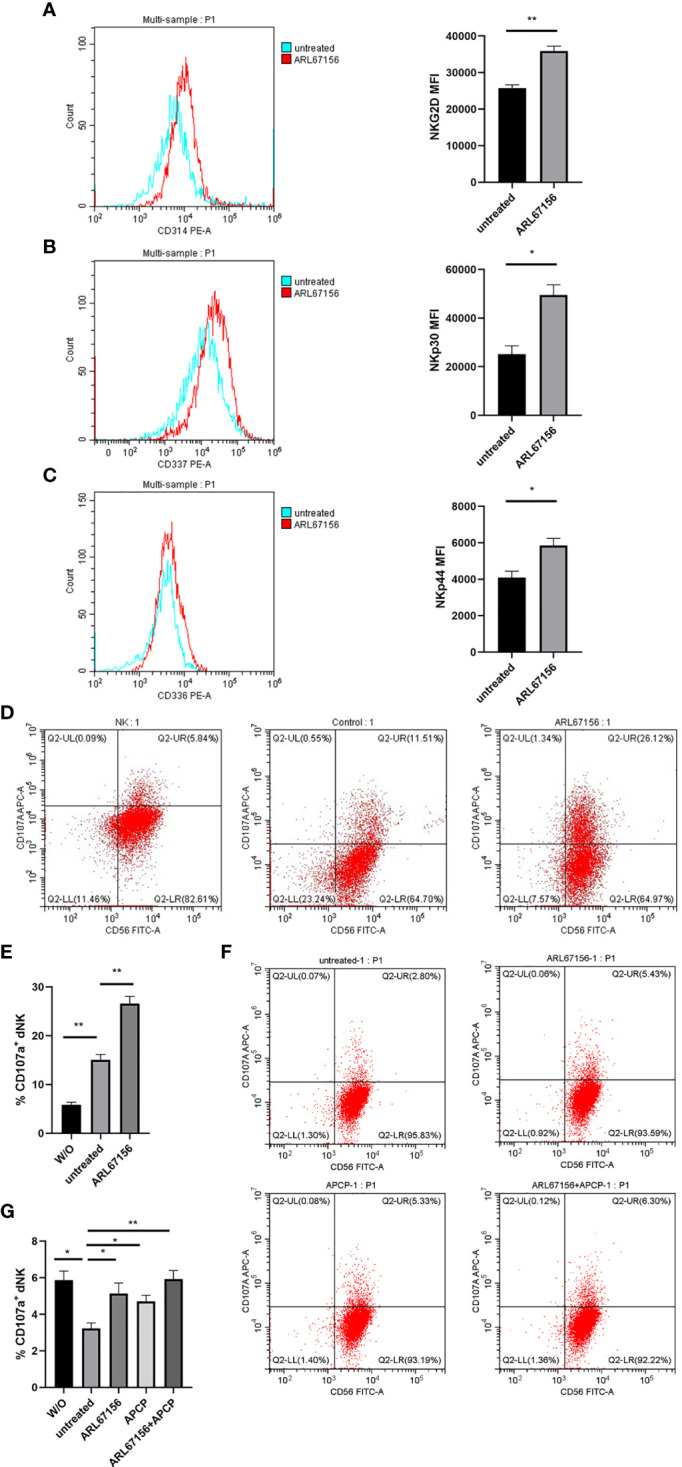
The cytotoxicity of dNK cells was regulated by CD39/CD73 enzyme activity. **(A–C)** Freshly isolated dNK cells from healthy donors were incubated in the absence or presence of the CD39 inhibitor ARL67156 for 24 hours, and then stained for the surface expression of the cytotoxicity markers NKG2D **(A)**, NKp30 **(B)** and NKp44 **(C)**. All experiments were performed in triplicate (mean ± SEM, *P < 0.05, **P < 0.01 by two-tailed t tests). **(D, E)** Effects of CD39 on the degranulation of activated dNK cells from healthy donors. To quantify degranulation, the surface expression of CD107a was measured after activation of isolated dNK cells were incubated with or without (W/O) the NK-susceptible target cell line (K562 cells) in the absence or presence of the CD39 inhibitor ARL67156 for 24 hours (mean ± SEM, **P < 0.01 compared with the untreated group, one-way ANOVA and *post hoc* tests). **(F, G)** dNK cells isolated from healthy donors co-cultured with HTR-8/SVneo cells (dNK : HTR-8/SVneo cells = 2:1) in the absence or presence of the CD39 inhibitor ARL67156 and/or CD73 inhibitor APCP for 24 hours. The percentage of dNK cells expressing CD107a was used as the indicator of degranulation (mean ± SEM, *P < 0.05, **P < 0.01, one-way ANOVA and *post hoc* tests).

We then investigated the effect of CD39 on the expression of dNK cell receptors. The expression of NKG2D, NKp30 and NKp44 on dNK cells isolated from normal pregnancies in the presence or absence of CD39 inhibitor for 24 hours was measured with flow cytometry. Increased expression of NKG2D, NKp30 and NKp44 in the dNK cells treated with the CD39 inhibitor was observed (**Figures 5A–C**).

Furthermore, flow cytometry was used to evaluate the CD107a (lysosomal associated membrane protein-1, LAMP-1) expression that reflects the cytotoxic degranulation ability of the dNK cells. The percentage of the activated dNK cells expressing CD107a was as low as 6.60% in the absence of other cells. However, after adding the NK-sensitive K562 cells, this percentage increased significantly to 15.1%. The percentage of CD107a^+^ dNK cells further increased to 28.7% when the CD39 inhibitor was added (**Figures 5D, E**), which indicated that CD39 reduces degranulation of the dNK cells.

Additionally, the expressions of CD107a on dNK cells co-cultured with the HTR-8/SVneo cells, in the presence or absence of the CD39 and/or CD73 inhibitor was investigated. Interestingly, the percentage of CD107a^+^ dNK cells decreased significantly to 2.83% when co-cultured with the HTR-8/SVneo cells, a result opposite to co-cultivation with the K562 cells. However, the percentage of dNK cells expressing CD107a in the co-culture system increased to approximately 5% in the presence of the CD39 or CD73 inhibitor, and returned to the original value of about 6% when both CD39 and CD73 inhibitors were added (**Figures 5F, G**). These results indicated that HTR-8/SVneo cells suppress the degranulation of dNK cells, and this suppression is associated with CD39 and CD73.

### Impacts of CD39/CD73 on the Invasion and Proliferation of HTR8/SVneo Cells

**Figure 6:**
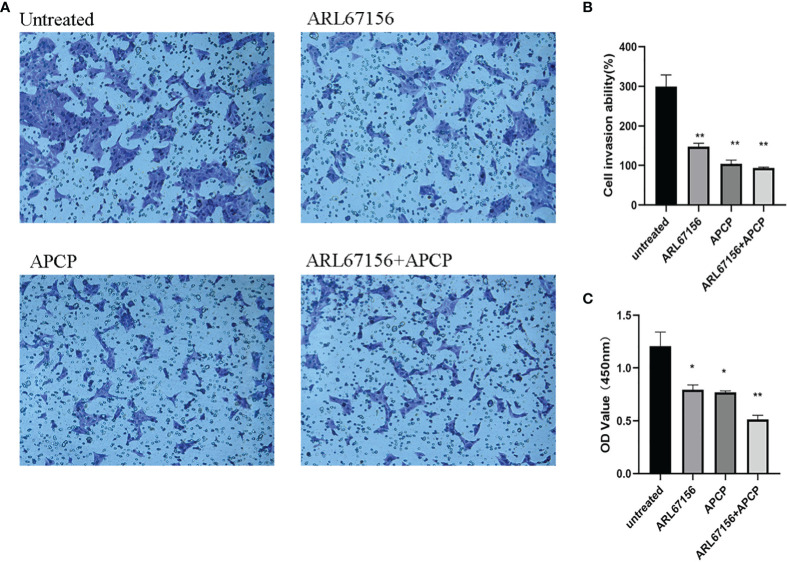
The change of invasion and proliferation of HTR8/SVneo cells after inhibiting CD39 and CD73. Representative photos **(A)** and data **(B)** for the matrigel transwell assay of normal dNK cells from healthy donors co-cultured with the HTR-8/SVneo cells (dNK: HTR-8/SVneo cells = 2:1) in the absence or presence of the CD39 inhibitor ARL67156 and/or the CD73 inhibitor APCP. n = 3 for each group (mean ± SEM, **P < 0.01 compared with the untreated group, one-way ANOVA and *post hoc* tests). **(C)** The OD values of the HTR-8/SVneo cells in the CCK-8 assay, showing their proliferation capacities (mean ± SEM, *P < 0.05, **P < 0.01 compared with the untreated group, one-way ANOVA and *post hoc* tests).

To evaluate changes in the invasion and proliferation abilities of the HTR8/SVneo cells, isolated dNK cells from normal pregnancies were co-cultured with HTR8/SVneo cells in the absence or presence of the CD39 inhibitor for 24 hours, and the matrigel transwell assay and CCK-8 assay were performed, respectively. It was found that the invasion of the HTR8/SVneo cells in the groups with CD39 or/and CD73 inhibitor was significantly less than that of the untreated group (**Figures 6A, B**). Compared with the untreated group, cell proliferation rates also fell in the CD39 or CD73 inhibitor groups, and the decrease was even more in the group exposed to both inhibitors (**Figure 6C**).

### TGF-β Induces the Expression of CD73 on HTR-8/SVneo Cells *via* the mTOR-HIF-1α Pathway

**Figure 7:**
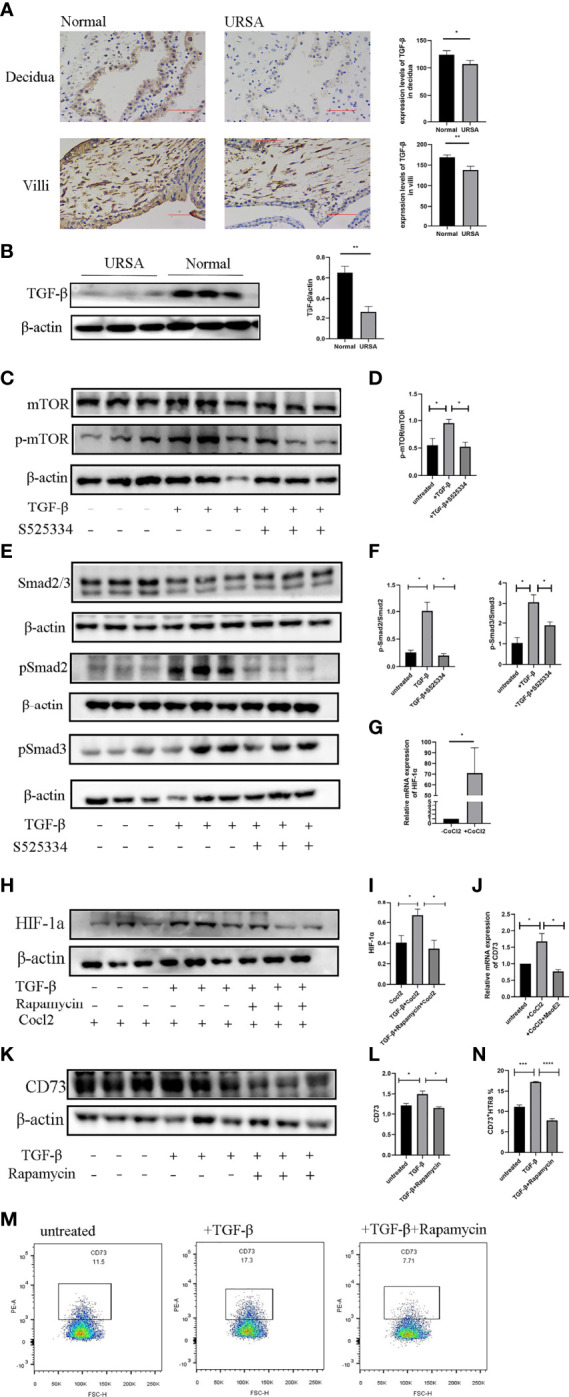
TGF-b induces the expression of CD73 on HTR-8/SVneo cells *via* mTOR-HIF-1a. **(A)** Immunohistochemical staining and villous tissues from normal and URSA pregnancies. Results are shown as mean ± SEM, n = 3 (*P < 0.05, **P < 0.01, two-tailed t tests). **(B)** Western blot analysis of TGF-β in the villous tissues from normal and URSA pregnancies. Results are shown as mean ± SEM, n = 3 (**P < 0.01, two-tailed t tests). **(C, D)** Western blot analysis of mTOR and pmTOR in HTR-8/SVneo cells cultured with or without rhTGF-β (10 ng/mL) and the Smad2/3 inhibitor S525334. **(E, F)** Western blot analysis of Smad2/3 and pSmad2 and pSmad3 in HTR-8/SVneo cells cultured with or without rhTGF-β (10 ng/mL) and the Smad2/3 inhibitor S525334. **(G)** Relative mRNA levels of HIF-1α in the HTR-8/SVneo cells cultured with or without CoCl_2_ (100 mM). **(H, I)** HTR-8/SVneo cells were treated with or without rapamycin (10 nM) for 1 hour, and then stimulated with rhTGF-β (10 ng/mL) (or vehicle) and CoCl_2_ (100 mM) for 12 hours. The whole cell lysate was analyzed for HIF-1α by Western blot. **(J)** HTR-8/SVneo cells were cultured with or without CoCl_2_ (100 mM) and the HIF-1α inhibitor MeoE2 (10 mM) for 24 hours. Relative mRNA levels of CD73 were measured by RT-PCR. **(K, L)** HTR-8/SVneo cells were treated with the mTOR inhibitor rapamycin (10 nM) for 1 hour and then with rhTGF-β (10 ng/mL) or vehicle for 24 hours. The whole cell lysate was analyzed for CD73 by Western blot. **(M, N)** The percentages of CD73^+^ HTR-8/SVneo cells from the abovementioned experiment were further analyzed with flow cytometry. Means of three different experiments ± SEM are shown (*P < 0.05, **P < 0.01, ***P < 0.001, ****P < 0.0001, two-tailed t tests or one-way ANOVA and *post hoc* tests).

We then explored the potential mechanism for the CD73 downregulation in URSA. Compared with the samples from normal pregnancies, the level of TGF-β in the decidual and villous tissues was significantly lower in the URSA pregnancies, as revealed by immunohistochemical staining (**Figure 7A**). Western blots showed the same result - the level of TGF-β in the villous tissue of URSA patients was significantly lower than that in tissues from normal pregnancies (**Figure 7B**).

To study whether and how TGF-β induces the expression of CD73 on the HTR-8/SVneo cells, we measured the levels of pmTOR (**Figures 7C, D**), the rapid phosphorylation products of downstream mTOR effectors pSmad2 and pSmad3 (**Figures 7E, F**), as well as CD73 (**Figures 7K–N**) in the HTR-8/SVneo cells after rhTGF-β treatment, and found increased expression of all the receptors and effectors. Moreover, these increases could be diminished by the addition of Smad2/3 inhibitor S525334, suggesting a TGF-β-mTOR signaling pathway in the HTR-8/SVneo cells.

We further analyzed the activation of HIF-1α, since it has been shown that CD73 is a direct target of HIF-1α (48). The hypoxia mimetic CoCl_2_ was used to increase the level of HIF-1α in the HTR-8/SVneo cells (**Figure 7G**). TGF-β enhanced HIF-1α expression in the HTR-8/SVneo cells in the presence of CoCl_2_ (**Figures 7H, I**). However, this TGF-β-induced HIF-1α expression was reduced by the addition of rapamycin. In addition, CoCl_2_ treatment enhanced the CD73 expression, which was abrogated by the HIF-1α inhibitor MeoE2 (**Figure 7J**). When the mTOR pathway in the HTR-8/SVneo cells was suppressed by rapamycin, the TGF-β-mediated expression of CD73 also decreased (**Figures 7K, L**). This observation was confirmed with flow cytometry analyses (**Figures 7M, N**). Thus, our data indicated that TGF-β may induce CD73 expression in the HTR-8/SVneo cells *via* the mTOR-HIF-1α pathway.

## Discussion

**Figure 8:**
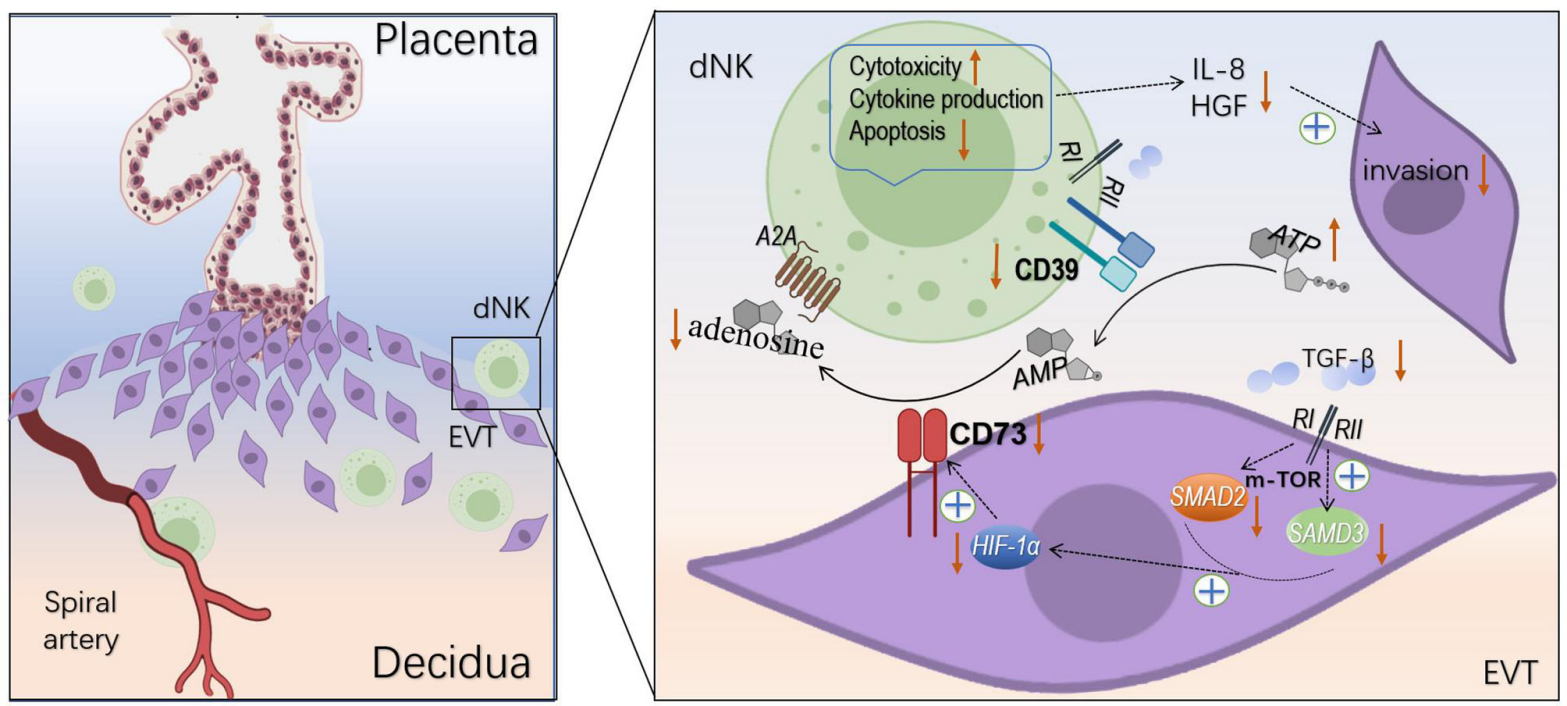
Regulation of dNK cells and EVT cells by CD39 and CD73. CD39 and CD73 hydrolyze ATP to adenosine changed dNK cells cytotoxicity, apoptosis, cytokine production, and impaired invasion of EVT cells. TGF-β induces the expression of CD73 on the EVT cells *via* mTOR-HIF-1α pathway.

In this study, we found marked decrease levels of CD39 and CD73 in the tissue from URSA patients compared with that from normal pregnancies. The decreases in CD39 and CD73 resulted in increased toxicity, decreased apoptosis, altered cytokine secretion of the dNK cells, as well as impaired invasion and proliferation of the co-cultured HTR8/SVneo cells, and may be caused by downregulated TGF-β-mTOR-HIF-1α pathway. To the best of our knowledge, this is the first study on CD39/CD73 at the maternal-fetal interface in URSA (**Figure 8**).

Three subgroups of dNK cells have been described, namely dNK1, dNK2 and dNK3 cells. All the three subgroups express CD49 and CD9. However, dNK1 cells also express CD39, CYP26A1 and B4GALNT1, dNK2 cells express ANXA1 and ITGB2, and dNK3 cells express CD160, KLRB1 and CD103, respectively. Studies have found that dNK1 cells contain more cytoplasmic granules than dNK2 and dNK3 cells. Also, dNK1 cells are more active in glycolytic metabolism and KIR gene expression, and produce higher levels of LILRB1 and cytoplasmic granule protein. This suggests a more critical crosstalk between the dNK1 and EVT cells (8). It could be speculated that CD39 on the surface of dNK1 may play a key role in communicating with EVT.

Both extracellular ATP and adenosine can induce apoptosis. ATP possibly induces apoptosis *via* activation of P2X7R (49, 50), while adenosine triggers apoptosis *via* A2A receptors. The potency of ATP and adenosine in induction of apoptosis may be different for different cells, depending on the receptors on the cell surfaces (51). For example, Wang, et al. found extracellular adenosine induces apoptosis of HGC-27 cells more strongly than ATP (52). In our study, A2A is present on dNK cells and hence the apoptosis may be triggered by adenosine rather than ATP. Extracellular cAMP may also be hydrolyzed to adenosine (53). However, no significant difference between cAMP concentration was observed between the groups (**Figure S3**), and hence the cAMP-adenosine pathway may not be involved in URSA. Therefore, it is likely that CD39 increases the level of intracellular adenosine and acts on A2A receptors, resulting in the increase of mitochondrial membrane potential and apoptosis of dNK cells.

The cytokine analyses showed that the dNK cells from URSA patients, which had lower levels of CD39, secreted higher levels of IL-10 than dNK cells from normal pregnancies. Samudra et al. have also shown that the production of IL-10 leads to miscarriage, which may be inhibited by CD39 (54). When CD39 was inhibited, dNK cells increased the production of IL-10 *in vitro*, suggesting a phenotypic shift to activated dNK cells (55). IL-10 is known as an anti-inflammatory cytokine and, in the majority of studies, CD39 is positively associated with IL-10 excretion (i.e. CD39 blockage reduces IL-10 levels). However, our study and the study by Samudra et al. (54) showed the opposite phenomenon, namely a negative association between CD39 and IL-10. This may be because that both studies investigated miscarriage associated with the maternal-fetal interface. Considering that NK cell-derived IL-10 is critical in the DC-NK cell crosstalk (56), and that dNK cells exhibit lower cytotoxicity but higher secretion potential, it is possible that the regulatory mechanism for IL-10 secretion in dNK cells at the maternal-fetal interface is different from other cells, although this hypothesis requires further investigation. *In vitro* and *in vivo* studies have both shown that IL-8 and IP-10 regulate trophoblast invasion (57). We found the secretion of IL-8 was significantly reduced after inhibiting CD39 in the co-culture system, but the secretion of IP-10 was not affected by either CD39 or CD73 inhibition, suggesting the regulation of IP-10 may not rely on CD39 or CD73. The secretion of IFN-γ by dNK cells transforms spiral arteries from arterioles that are constricted, muscular, and vasoactive to vein-like structures with dilated thin-walls (58), but our data showed that CD39 has no regulatory effect on IFN-γ secretion by dNK cells. HGF promotes the trophoblast cells incorporation into the walls of endothelial tubes, indicating a role in the differentiation of trophoblast intravascular and the remodeling of vascular during pregnancy. HGF derived from dNK cells is likely to act as a paracrine factor of the decidua, guiding the differentiation of trophoblast cells along the invasion pathway (59). As expected, inhibition of CD39 reduced HGF secretion. In addition, our data showed that CD39 inhibitors reduced the invasion and proliferation of the HTR-8/SVneo cells (**Figure 6**). Taken together, the cytokine data suggest that CD39^+^ dNK may promote the production of IL-8 and HGF to support the continuous invasion of EVT and the transformation of uterine spiral arteries.

Generally, dNK cells display lower cytotoxicity and higher cytokine secretion than pNK cells, which prevents immune attack of the fetal cells (7). Previous studies have shown that NKP30, NKP44 and NKG2D can increase the cytotoxicity of circulating NK cells *via* binding to related ligands (60, 61). Our results showed reduced expression of NKP30, NKP44 and NKG2D in CD39^+^ dNK cells. It was likely that the loss of CD39 may transform dNK cells into a highly toxic state by promoting the expression of cytotoxicity receptors. In the co-cultivation experiments, the combined effects of CD39 and CD73 inhibited the cytotoxicity of dNK cells. Interestingly, HTR8/SVneo cells also showed the ability to suppress dNK cytotoxicity *in vitro*. Studies have shown that exogenous TGF-β can stimulate the expression of CD39 and CD73 in T cells and dendritic cells (62, 63). In this study, we found the villous tissues of URSA patients displayed lower level of TGF-β compared with the normal pregnancies. In the subsequent *in vitro* experiments, we found that TGF-β stimulated the CD73 expression in the HTR-8/SVneo cells. After adding exogenous TGF-β into the culture system, pSmad2, pSmad3 and pmTOR signals increased simultaneously in the HTR-8/SVneo cells. This observation indicated a link between Smad signaling and mTOR activation in the HTR-8/SVneo cells. We also found that the Smad2/3 inhibitor SB525334 inhibited TGF-β-induced phosphorylation of mTOR, which suggested that this phosphorylation was Smad2/3-dependent. In addition, we proved that the activation of HIF-1α induced by exogenous TGF-β is mTOR-dependent, and is essential for the induction of CD73 under normoxic conditions. On the contrary, mTOR activity is not required in hypoxia-induced HIF-1α activation. These results indicate that the TGF-β-mTOR-HIF-1α pathway has important significance in regulating immune tolerance *via* CD73. In addition, we found that the decidual tissue of URSA patients showed lower level of TGF-β, so it was possible that TGF-β may regulate the expression of CD39 in dNK cells in the same way.

Some limitations exist in this study. Firstly, although we found extracellular adenosine activated the dNK cells, which receptor the adenosine activated was uncertain. Among the four types of purinergic receptors (A1, A2A, A2B and A3), A2A is the one highly expressed on lymphocytes, and the immune regulation of NK cells by adenosine *via* the purinergic receptor A2A has been extensively reported (24, 64–66). In addition, activation of A2B receptors requires a high concentration of adenosine, which is not common in physiological conditions (67). The cAMP/protein kinase A (PKA)/cAMP response element binding protein (CREB) signaling pathway is positively regulated by adenosine acting on A2A receptor (53, 68–70). We believe the effects of adenosine on the dNK cells were also A2A-dependent. It was possible that CD39 increases the level of intracellular cAMP by increasing the level of extracellular adenosine and acting on A2A receptor, resulting in the subsequent effects of dNK cells. Secondly, although we observed reduced level of TGF- β in URSA, neither the source or the cause was revealed. The decidual tissue is rich in TGF-β (71), which may be excreted by many cell types including decidual stromal cells (72), NK cells (41, 73), and trophoblasts (74). This study focused on the regulatory pathway from TGF-β to adenosine, and hence the upstream and downstream mechanism may be investigated in future studies. Thirdly, only limited quantities of decidual tissue was obtained due to practical reasons, hence limiting the numbers of replicates for certain analyses. Fourthly, although HTR8/SVneo cells are often used as substitutes for EVT, they cannot fully represent EVTs under physiological conditions. Lastly, animal studies were not conducted in this study due to lack of an ideal model, although the clinical samples and the isolated dNK cells co-cultured with HTR8/SVneo cells provided sufficient evidence to support the major findings of this study.

The pathway found in this study may be an important immunoregulatory mechanism that causes URSA, and provides a potential new therapeutic target for the prevention of URSA. However, the cause for the downregulated TGF-β in URSA and the detailed signaling pathways within dNK cells in response to extracellular adenosine require future exploration.

## Data Availability Statement

The raw data supporting the conclusions of this article will be made available by the authors, without undue reservation.

## Ethics Statement

The studies involving human participants were reviewed and approved by Ethics Committee of Chongqing Medical University. The patients/participants provided their written informed consent to participate in this study. Written informed consent was obtained from the individual(s) for the publication of any potentially identifiable images or data included in this article.

## Author Contributions

JZ wrote the original draft preparation. JZ, GS and XZ designed the experiment and method. BN, PB, T-LH and TM curated the data. JZ, HC and XY prepared the figures and collected the samples. CC, RC, RS, and HZ reviewed the data and the manuscript. All authors reviewed and approved the final manuscript.

## Funding

This research was supported by National Natural Science Foundation of China (No. 81971406, 81771607, 81871185, 81961128004, 81701477, 82071671), The 111 Project (Yuwaizhuan (2016)32), The National Key Research and Development Program of Reproductive Health & Major Birth Defects Control and Prevention (2016YFC1000407), Chongqing Health Commission (2017ZDXM008, 2018ZDXM024), and Chongqing Science & Technology Commission (cstc2017jcyjBX0060, cstc2018jcyjAX0359).

## Conflict of Interest

The authors declare that the research was conducted in the absence of any commercial or financial relationships that could be construed as a potential conflict of interest.

## Publisher’s Note

All claims expressed in this article are solely those of the authors and do not necessarily represent those of their affiliated organizations, or those of the publisher, the editors and the reviewers. Any product that may be evaluated in this article, or claim that may be made by its manufacturer, is not guaranteed or endorsed by the publisher.

## Abbreviations

URSA, unexplained recurrent spontaneous abortion; dNK, decidual natural killer; pNK, peripheral blood natural killer; EVT, extravillous trophoblast; TGF-β, transforming growth factor-β; mTOR, mammalian target of rapamycin; HIF-1α, hypoxia-inducible factor-1α.
