# Supplementary material for: CD39/CD73 Dysregulation of Adenosine Metabolism Increases Decidual Natural Killer Cell Cytotoxicity: Implications in Unexplained Recurrent Spontaneous Abortion
Source: Front Immunol. 2022 Feb 10;13:813218. doi: 10.3389/fimmu.2022.813218 (PMC8866181; doi:10.3389/fimmu.2022.813218)
Supplement: Supplementary file 1 [file DataSheet_1.pdf]

Supplementary Material

Table S1. Characteristics of the patients and controls

	Normal	URSA
Sample size	30	12
Age	24.3±0.41	24.8±0.59
Pregnant weeks	7.83±0.19	7.60±0.25
Numbers of abortion	1.3±0.16	2.6±0.15

Data are presented as mean ± SEM. No significant difference in age and pregnant weeks was observed between the two groups. Comparison of numbers of abortion between the two groups was not conducted.

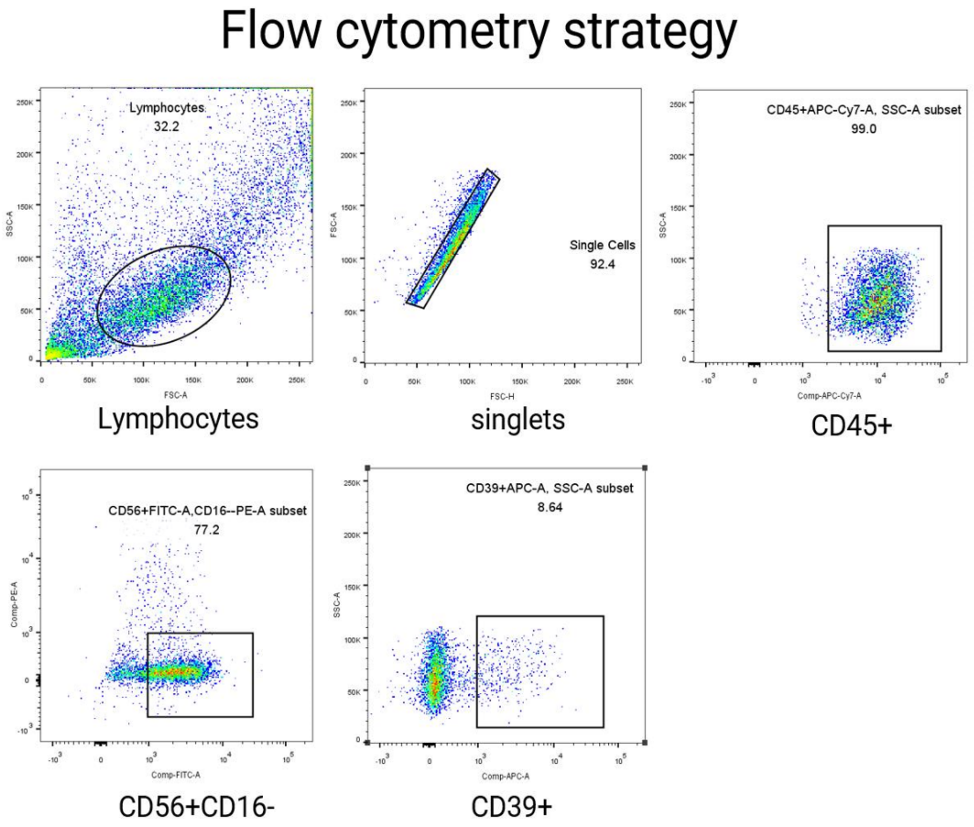

Figure S1. Gating strategy of the multidimensional flow cytometry analysis. The representative scatter diagrams show the gating strategy for flow cytometric analysis

of dNK cells from normal decidual tissue. Following singlet events gating (FSC_A and FSC_H), viable CD45+ lymphocytes were identified. The CD56+CD16-dNK population and phenotype were examined according to the expression of surface marker CD39+dNK (NKp44, NKp30, NKG2D and CD107a).

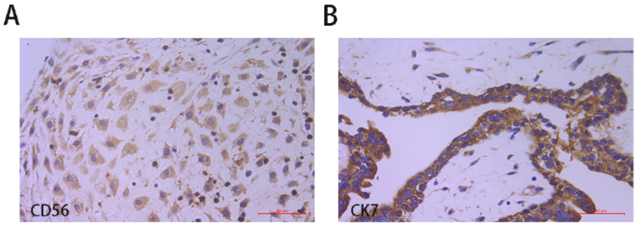

Figure S2. Immunohistochemical staining of CD56 and CK7 in the decidual and villous tissues. Representative photographs of CD56 (**A**) and CK7 (**B**) immunohistochemical staining in normal decidual and villous tissues, respectively. Scale bar =50 μm.

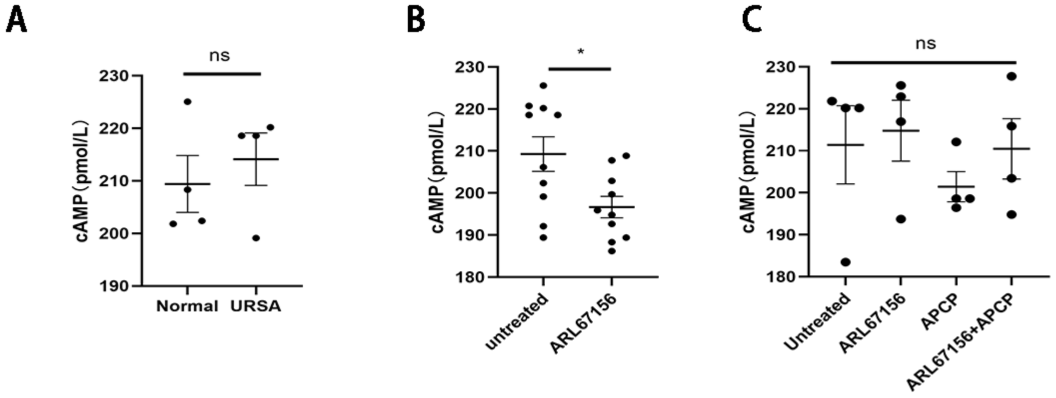

Figure S3. Production of cAMP in dNK cells. (**A**) dNK cells were isolated from decidual tissues of normal pregnant women (n=4) and URSA patients (n=4), and then cultured in a 24-well plate (2×10^5 cells/well) for 24 hours. The concentration of cAMP in the media was measured using ELISA (mean ± SEM, two-tailed t test). (**B**) Freshly

prepared dNK cells (n=10) were isolated from healthy decidua and incubated in the absence or presence of CD39 inhibitor (ARL67156) in a 24-well plate (2×10^5 cells/well) for 24 hours. The concentration of cAMP in the media was measured using ELISA (mean ± SEM, $^*P < 0.05$, two-tailed t test). (**C**) dNK cells isolated from healthy decidua (n=4) were co-cultured with HTR-8/SVneo cells (dNK cells:HTR-8/SVneo cells = 2:1) and incubated in the absence or presence of the CD39 inhibitor ARL67156 and/or the CD73 inhibitor APCP in a 24-well plate (2×10^5 cells/well) for 24h. The concentration of cAMP in the media was measured using ELISA (mean ± SEM, compared with the untreated group, one-way ANOVA and post hoc tests).
